# Improvement of the surgical curability of locally confined prostate cancer including non-organ-confined high-risk disease through retropubic radical prostatectomy with intentional wide resection

**DOI:** 10.1186/1477-7819-10-249

**Published:** 2012-11-16

**Authors:** Eijiro Okajima, Motokiyo Yoshikawa, Yasumasa Masuda, Kazuhiro Shimizu, Nobumichi Tanaka, Akihide Hirayama, Keiji Shimada, Kiyohide Fujimoto, Yoshihiko Hirao

**Affiliations:** 1 Department of Urology, Nara City Hospital, Eijiro Okajima Higasikidera-cho 1-50-1, Nara, Nara, 630-8305, Japan; 2 Kashiwai Clinic, Eijiro Okajima Higasikidera-cho 1-50-1, Nara, Nara, 630-8305, Japan; 3 Department of Urology, Nara Medical University, Eijiro Okajima Higasikidera-cho 1-50-1, Nara, Nara, 630-8305, Japan; 4 Department of Pathology, Nara Medical University, Eijiro Okajima Higasikidera-cho 1-50-1, Nara, Nara, 630-8305, Japan

**Keywords:** Prostate, Neoplasms, Prostatectomy

## Abstract

**Background:**

Retropubic radical prostatectomy with intentional wide resection (RRP-WR), which enables clear location of the prostate apex and the performance of posterolateral wider resection to remove extraprostatic extension, was introduced to our institutions. The aim of this study is to assess the feasibility and the efficacy of RRP-WR as a surgical intervention for locally confined prostate cancer.

**Methods:**

A total of 90 Japanese patients with pathologically proven and clinically locally confined hormone-naïve prostate cancer were treated through RRP-WR, and the surgical morbidity was assessed. The patients were observed without immediate treatment until biochemical recurrence (BCR).

**Results:**

The surgical morbidities were comparable to conventional procedures. No positive surgical margin (pSM) was pathologically identified in pT2 cases from prostatectomy specimens. It was identified in only 14.3% of pT3a cases, 36.4% of pT3b cases and 100% of pT4 cases. No apical pSM was found except for one of the pT4 cases in the levator ani muscle. PSA was at an undetectable level in 80.0% of all cases, 90.0% of pT2 cases, and 67.5% of pT3 and pT4 cases after surgery. The BCR-free survival rate in all cases was 82.4% and that of high-risk cases without pSM was 76.9% at a median follow-up of 19.3 months (3.3 to 59.2).

**Conclusions:**

RRP-WR is feasible and effective in removing organ-confined prostate cancer as well as extraprostatic extension without pSM. Thus, it is worthwhile to evaluate if this procedure improves the clinical outcome of locally confined prostate cancer including high-risk conditions treated by surgical intervention.

## Background

The advent of prostate-specific antigen (PSA) screening has resulted in an overall reduction of prostate cancer. It has also been found that the majority of prostate cancer cases are diagnosed in individuals over 60 years old and grow slowly, meaning that they are not lethal within the remaining life span. However, there are still cases in which clinically locally confined but high-risk prostate cancer progresses and causes metastasis and cancer death [1]. The benefits of the various treatment options available for those with high-risk disease remain unproven [2]. Even though radical prostatectomy results in excellent prognosis for low-risk prostate cancer [3], for high-risk cases, the guidelines of the European Association of Urology (EAU) [4] and the National Comprehensive Cancer Network (NCCN) [5] recommend the procedure as an optional therapy.

The goal of radical prostatectomy for locally confined prostate cancer is complete prostate extirpation, and it is well known that curable resection without a positive surgical margin (pSM) is essential for positive results [6]. Since high-risk locally confined prostate cancer often demonstrates extraprostatic extension (EPE) in prostatectomy specimens exhibiting pSM, better prognostic results can be expected from a wider resection than that of the current operative procedure. Since more than half of pSM is found in the apex of the prostate compared to its other parts [7], it is also important to clearly locate the prostate apex.

Fujimoto reported on a method of intentional wide resection for the prostate that enables clear location of the prostate apex and the performance of posterolateral wider resection to remove EPE in cases without pSM [8-10].

In this study, to improve the control of locally confined prostate cancer, we evaluated retropubic radical prostatectomy with intentional wide resection (RRP-WR) – a procedure that can not only remove some of the EPE of the cancer but can also reduce the apical pSM – for locally confined prostate cancer, including high-risk cases.

## Methods

We diagnosed prostate cancer pathologically using more than eight biopsy specimens per individual and ascertained the stage of locally confined disease through bone scanning and computed tomography and/or magnetic resonance imaging. After excluding cases with cT3b (seminal vesicle involvement) and worse (cT4) local-stage development through imaging study (because Fujimoto et al. reported a poor survival benefit from RRP-WR even with neoadjuvant androgen-deprivation therapy [10]), we gave informed consent in terms of treatment for locally confined prostate cancer, including active surveillance, brachytherapy, external beam radiation, intensity modulated radiation therapy, and radical prostatectomy with or without nerve preservation. From January 2007 to December 2011, 90 prostate cancer patients (cT1c, cT2 or cT3a) who accepted radical prostatectomy were treated with RRP-WR without any prior therapy, and we observed them without immediate post-surgical adjuvant therapy. Three patients had unilateral neurovascular bundle preservation and contralateral wide resection. The clinicopathological characteristics of all 90 cases before surgery are shown in Table 1.

**Table 1:** Patient characteristics

	**All 90 cases**	**High**-**risk cases (32)**
Age (years)	68.1 (54 – 78)	68.1 (57 – 78)
Initial PSA (ng/ml)	11.39 (3.12 – 33.93)	17.10 (4.72 – 33.93)
Gleason score in biopsy specimens	7.0 (6 – 9)	7.5 (6 – 9)
Maximum cancer occupation per a biopsy core (%)	42.4 (5 – 100)	52.4 (5 – 100)
Gleason score in prostatectomy specimens	7.1 (6 – 9)	7.5 (6 – 9)
Follow-up period after surgery (months)	23.0 (3.3 – 59.2)	17.4 (3.3 – 56.1)

High-risk cases were defined as those with a Gleason score of 8 to 10, an initial PSA of more than 20 ng/ml or a preoperative local stage of cT3a according to the criteria of NCCN [5,11]. There were 32 cases of high-risk prostate cancer.

After lymph node dissection of the bilateral obturator area, we performed intended wide resection according to the method outlined by Fujimoto et al. [8-10]. Briefly, after incision of the endopelvic fascia along the pelvic wall, we identified the neurovascular bundle before performing incision of the lateral pelvic fascia posterolaterally from the neurovascular bundle. We then separated the mesorectal fat from the muscle layer of the rectum until it could no longer be easily separated to the point at which the rectourethral muscle was reached or until the proximal region of either the vas deferens or the peritoneum of the Douglas fossa was identified. Knowing the position of the external rectal sphincter clarifies the location of the urethral sphincter.

A single pathologist (K.S.) evaluated the degree of malignancy in the biopsy and prostatectomy specimens according to the Gleason grading system [12] and the stage based on the 2004 TNM classification [13]. The prostatectomy specimens were fixed in 10% buffered formalin and sectioned into 3-mm slices in the plane perpendicular to the long axis of the gland from the apex to the tip of the seminal vesicles, followed by hematoxylin and eosin staining and determination of EPE. Eighteen cases (32.1%) of 56 cT1c cases and 11 cases (64.7%) of 17 cT2a cases were found pT3 or pT4 (Table 2).

**Table 2:** Number of cases with positive surgical margin according to preoperative clinical and pathological T category

	**cT1c**	**cT2a**	**cT2b**	**cT3a**	
pT2a	12	3	0	0	15
pT2b	8	1	2	0	11
pT2c	18	2	0	4	24
pT3a	16 (pSM*: 2)	6 (pSM*: 2)	0	6	28 (pSM*: 4)
pT3b	2	4 (pSM*: 2)	1	4 (pSM*: 2)	11 (pSM*: 4)
pT4	0	1 (pSM*: 1)	0	0	1 (pSM*: 1)
	56 (pSM*: 2)	17 (pSM*: 5)	3	14 (pSM*: 2)	90 (pSM*: 9)

pSM: positive surgical margin.

We analyzed factors associated with surgical morbidity, such as operative time, blood loss during the operation, number of days with a urethral catheter after surgery, number of days in the hospital after surgery and any adverse events.

After prostatectomy, we followed up with a PSA assay every 3 months for the first 2 years, every 6 months for the next 3 years, and yearly thereafter. We also added PSA measurement if unfavorable PSA elevation was observed. The mean and median follow-up values were 23.2 and 19.3 months (range: 3.3 – 59.2), respectively.

Even though the efficacy of cancer treatment should be evaluated by cause-specific survival, we used the achievement of undetectable PSA, which has been reported as a significant predictor of biochemical recurrence (BCR) in pT3 cases after surgery by multivariate analysis [14], and BCF-free survival as surrogate endpoints because the natural history of prostate cancer is very long and PSA is extremely sensitive. The date of BCR was defined as the time at which the serum PSA level exceeded 0.2 ng/ml after surgery. The patients received no adjuvant therapy immediately after surgery, and they were subsequently treated with radiation therapy and/or androgen deprivation therapy only after BCR as described above. Four cases with lymph node metastasis were found among the 90 cases included.

We used the Kaplan-Meier survival estimation curve and the log rank method for univariate analysis. As the number of cases was relatively small, it was not possible to perform multivariate analysis to find factors for predicting the clinical outcome of RRP-WR.

## Results

### Morbidity with RRP-WR

The mean and median operative times were 307 min and 304 min, respectively. It took over 5 h to perform the operation for the first year, as it was a newly introduced procedure. After around 20 cases, our technique had advanced to enable completion within 4 h.

The mean and median blood loss amounts during surgery were 998 ml and 858 ml, respectively. Auto-transfusion covered the bleeding, and no allo-transfusion was given to any of the patients. As the operating time decreased, blood loss decreased.

In half the cases the urethral catheter was removed following initial cystography 4 or 5 days after surgery. The mean and median numbers of days with catheter presence were 10.2 and 8, respectively.

The number of days in the hospital after surgery depended on the progress of surface wound healing rather than on healing related to urethral anastomosis. Half the patients left the hospital less than 9 days after surgery. The mean and median numbers of days in the hospital after surgery were 11.3 and 8, respectively. Incontinence requiring more than two pads a day was observed 3 months after surgery in six cases (6.5 %). After 6 months, incontinence requiring more than one pad a day was seen in only three cases (3.8% of 80 cases, with follow-up lasting more than 6 months) with early BCR.

Intentional wide resection aimed to reduce the pSM rate, which by its nature cannot preclude an outcome of erectile dysfunction. In this work, we did not assess erectile dysfunction because it is obvious that patients will suffer loss of erectile functionality when given intentional wide resection.

Three had sustained lymph leakage, three developed stricture at urethral anastomosis, two had a wound infection, one had bleeding 2 days after surgery, one had a minor pulmonary infarction, and one patient with a past history of urolithiasis had unilateral hydronephrosis of unknown origin that improved spontaneously (Table 3).

**Table 3:** Adverse events

**Event**	**Number of cases**
Lymph leakage	3
Stricture at urethral anastomosis	3
Wound infection	2
Bleeding 2 days after operation	1
Pulmonary infarction	1
Hydronephrosis	1
Incontinence requiring more than 1 pad a day*	3

*Among 80 cases, with follow-up lasting 6 months.

### Pathological results of 90 cases from prostatectomy specimens

Eighty of all 90 cases demonstrated perineural invasion. Gleason scores of 7 were found in 74 cases (82.2%), and scores of 9 were found in 6 cases (6.7%). Two patients with pT2c, one with pT3a and one with pT3b had lymph node metastasis. One patient was diagnosed with pT4 with levator ani invasion.

In terms of the association of preoperative clinical local staging with pathological results in prostatectomy specimens, we adopted cases in this study to the Japan PC table [15]: a preoperative nomogram for Japanese patients with locally confined prostate cancer. There were 58 cases clinically diagnosed as organ-confined (T1 and T2) and low risk by NCCN criteria. According to the Japan PC table, the probability of EPE for 34 of 58 cases was less than 25%, and 9 of these 34 (26.5%) patients were found to have EPE by prostatectomy specimens. And 24 of 58 patients were predicted to have a more than 25% probability of EPE, and 13 of these 24 (54.2%) patients were found to have EPE. Eighteen cases were clinically diagnosed as organ-confined (T1 and T2) and high risk by the NCCN criteria. The probability of EPE for 15 of 18 (83.3%) cases was over 25%, the mean probability of EPE for these 18 cases was 41.6%, and 7 of 18 cases (38.9%) were found to have EPE by prostatectomy specimens.

No pSM was found in 50 cases of pT2, and only 4 cases (14.3%) with pSM were found in 28 cases of pT3a. Even among 11 cases of pT3b, 7 were removed without pSM.

### Preliminary clinical outcome of 90 cases by PSA

PSA was reduced to undetectable levels in 72 (80.0%) of 90 cases. BCR was found in 15 cases during the follow-up period (Table 4).

**Table 4:** pSM and PSA decline according to pathological T category

**Pathological T**		pSM* (%)	**Undetectable PSA(%)**
T2a	15	0 (0)	15 (100)
T2b	11	0 (0)	8 (72.7)
T2c	24	0 (0)	22 (91.7)
T3a	28	4 (17.9)	19 (67.9)
T3b	11	4 (36.4)	8 (72.7)
T4	1	1 (100)	0 (0)
Total	90	9 (10)	72 (80)

pSM: positive surgical margin.

BCR-free survival at a median follow-up of 19.3 months (3.3-59.2) was estimated at 82.4% using the Kaplan-Meier survival estimation curve, with 23.0 months (range: 3.3 – 59.2) of mean follow-up for the 90 patients.

**Figure 1:**
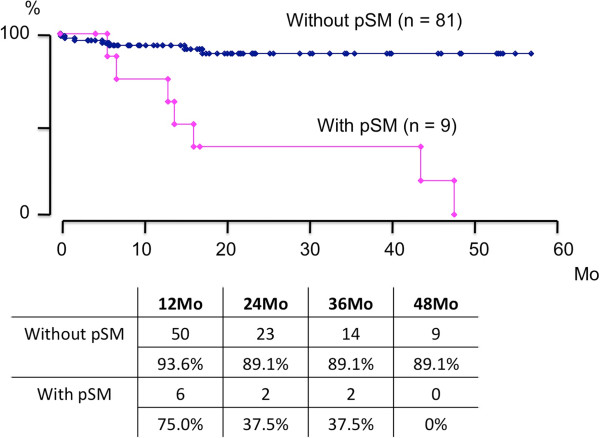
**BCR-free survival rate in cases with and without pSM from prostatectomy specimens.** BCF-free survival rates in cases with and without pSM were shown to be 37.5% and 89.1% at 2 years estimated to achieve BCR-free survival, respectively.

**Figure 2:**
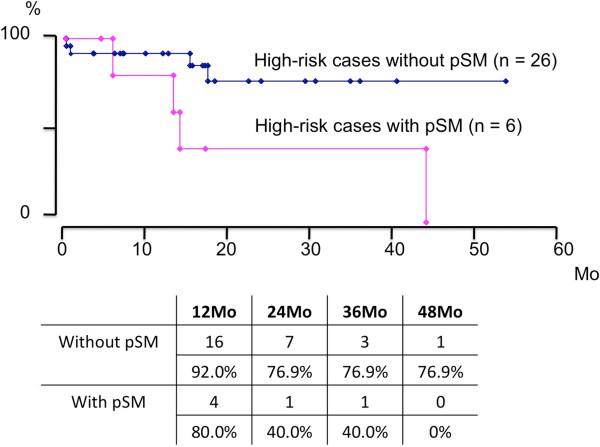
**Estimated BCR-free survival rate difference in cases with and without pSM among 18 high-risk cases treated with RRP-WR alone among 32 high-risk cases;BCR-free survival rates in cases with and without pSM was shown as being 40.0%and 76.9%at 3 years estimated to achieve BCR-free survival,respectively.**

Although we did not compare inter-group difference statistically because the follow-up period is too short for prostate cancer, BCR-free survival rates in cases with and without pSM were demonstrated as being 37.5% and 89.1% of those that were estimated at 2 years to achieve BCR-free survival, respectively (Figure 1). Furthermore, among 32 high-risk cases, BCR-free survival rates in patients with and without pSM were demonstrated as being 40.0 % and 76.9% of those that were estimated at 2 years to achieve BCR-free survival, respectively (Figure 2).

**Figure 3:**
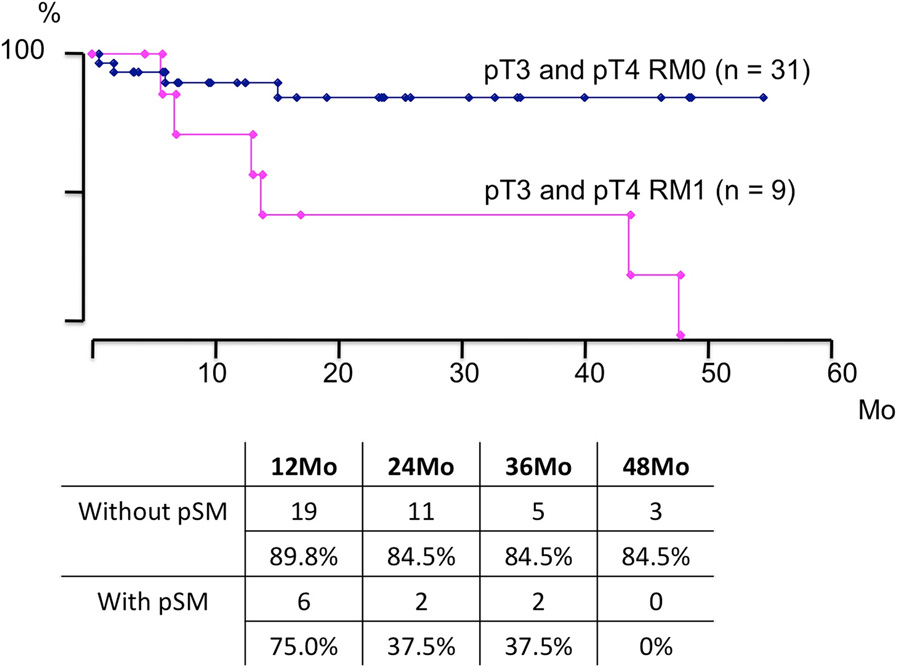
**Estimated BCR-free survival rate in cases with and without pSM among 40 pT3 and pT4 cases treated with RRP-WR alone.** BCR-free survival rates in cases with and without pSM were shown as being 42.9% and 84.5% at 3 years estimated to achieve BCF-free survival, respectively.

When focusing on cases of pT3 and pT4, there were 40 cases among the 90 patients. BCR-free survival of these 40 cases was estimated to be 74.3% at 2 years after surgery. BCR-free survival rates in cases with and without pSM were 42.9% and 84.5% of those that were estimated at 3 years to achieve BCR-free survival, respectively (Figure 3).

## Discussion

To improve prostate cancer control, we performed RRP-WR for 90 cases of preoperative locally confined prostate cancer, and we evaluated the surgical outcome by morbidity and curability with surgical margin status pathologically. We tried to demonstrate preliminary clinical outcomes by achievement of undetectable PSA and BCR without immediate adjuvant therapy after surgery.

Half the procedures took more than 4 h to complete, partly because of the time taken for lymph node dissection. We routinely performed obturator lymph node dissection, and internal iliac lymph node dissection was also implemented for high-risk cases by NCCN classification. Lymph node dissection in radical prostatectomy is mainly for the purpose of staging, but for advanced cases it may be therapeutic [16]. Only four instances of lymph node metastasis were found in this series of cases, and three of these four patients achieved undetectable postoperative PSA, two without BCR at 3 and 30 months, and the other two cases recurred biochemically at 25 and 47 months after surgery.

Although blood loss was not trivially small, it could be deemed acceptable because no allo-transfusion was required and the loss level of less than 1,000 ml is lower than or comparable to the values of 800 to 1,650 ml noted in previous reports [17,18]. The number of days with urethral catheter presence after surgery and the number of days spent in the hospital after surgery were also not longer than those in previous reports. It takes time to recover from incontinence (6.5% required more than two pads a day at 3 months after surgery), but after 6 months, incontinence with more than one pad a day was seen only in three cases with early BCR. Another report on this procedure also mentioned that a period of 6 months is required to recover continence [9]. Thus, the procedure can be seen as feasible.

Kupelian et al. reported that intracapsular pSM for organ-confined (pT2) disease from radical prostatectomy with inadvertent incision through the capsule in a tumor was 18% [19]. Kordon et al. also reported that among 1,378 cases of pT2, 16.9% showed pSM [20]. Even with minor modification to reduce apical pSM, pSM in organ-confined disease could not be eliminated completely [21]. In this study, we achieved resection without pSM for all pT2 patients through RRP-WR, although the number of cases was small. Locating the rectourethral muscle enabled clarification not only of the urethral sphincter’s location, but also that of the prostate apex. This is a major advantage of the procedure for resection without pSM. However, most cases with organ-confined disease are in a good risk group for which more than 10 years of survival can be expected with any definitive monotherapy [1,22]. In terms of control for organ-confined (pT2) prostate cancer, we consider that this procedure would be as successful as the regular RRP procedure, although more would cause erectile dysfunction, which was not analyzed in this work.

For patients with pT3 and pT4, curative resection was achieved without pSM at a rate of 77.5%. Inagaki et al*.* reported a no-pSM rate of 38.7% for 106 cases of pT3a and pT3b that had been diagnosed as cT1 and cT2 preoperatively [14], and Kordon et al. also reported that the incidence of no pSM in 288 pT3a cases was 52.1% [20], which is much lower than the respective 82.1% and 85.7% values seen in our series.

This increase in the ratio of resection without pSM for cases with pT3 and pT4 seems to have contributed to better cancer control. In this series, pT3 and pT4 cases without pSM demonstrated an 84.5% BCR-free survival rate 2 years after surgery, which is much higher than the 37.5% value seen for cases with pSM. Since the follow-up period is short, we also evaluated achievement of undetectable PSA after surgery, which has been reported as a significant predictor of BCR in pT3 cases after surgery by multivariate analysis [14], and found a figure of 92.5% for cases with pT3 and pT4. Recently, Preston et al. reported the prognostic significance of curative resection for cases with non organ-confined disease by radical prostatectomy [23]. They demonstrated that cases with completely resected extraprostatic disease had a higher probability of BCR-free survival (86 % at 5 years after surgery) than those with capsular incision into tumors of organ-confined disease did (77 % at 5 years after surgery). Thus, with RRP-WR we can expect better prognosis of locally confined prostate cancer with EPE treated surgically than previous reports indicate, although it is necessary to confirm the long-term results for a large number of cases before making a firm conclusion.

In terms of the indication of this procedure compared to regular RRP, we consider that locally confined but non-organ-confined or no seminal vesicle involvement (pT3a) cases may deserve the advantage of the procedure as opposed to regular RRP. As mentioned above, for good risk cases [such as those with an initial PSA of less than 10, a Gleason score of less than 7 or organ-confinement (pT2)], the regular and well-established procedure of radical prostatectomy is good enough to achieve a survival period of 10 years. The authors demonstrated 10-year cause-specific survival greater than 95% for pT2 cases treated by radical prostatectomy [24]. Erectile dysfunction, which cannot be avoided because of the nature of wide resection, is one disadvantage of this procedure, although we did not assess erectile dysfunction in this work. Thus, for pT2 cases, nerve-sparing prostatectomy results in a better risk/benefit ratio. Cases of pT3b (seminal vesicle invasion) and pT4 (direct invasion to surrounding organ or muscle) had poor outcomes even after resection without pSM from pathological evaluation of prostatectomy specimens [10]. As preoperative evaluation cannot ascertain beyond doubt whether the disease is pT3a, such cases cannot be chosen before the operation. Without pathological evaluation of the entire prostate after removal by radical prostatectomy, it is also not possible to tell at the preoperative stage whether the disease is organ-confined.

In this series, concerning the T category, we found that most of the cases (62 of 90 cases) were underestimated preoperatively compared to the results from the prostatectomy specimens. We reevaluate the cases of this study using the Japan PC table [15] to find the reason for this discrepancy. As the Japan PC table and NCCN risk criteria seem to correlate with each other in this series, our results are also in line with the prediction of the table. Thus, we confirmed that simple clinical T category cannot tell the pathological results in prostatectomy specimens and that the Japan PC Table is useful to determine the probability of EPE of clinically organ-confined disease.

Also in our series, 30 (40.5%) of 74 cT1c and cT2 cases were pT3 and worse pathologically. Among these 30 preoperatively underestimated cases, successful removal was achieved in 23 (76.7%) by RRP-WR. More than half of the cases in this series did not require RRP-WR to achieve resection without pSM, but 23 of 74 cases (31.1%) with cT1c and cT2 would have failed if the procedure had not been performed. Although it is difficult to expect a good prognosis for cases with pT3b and pT4 from radical prostatectomy alone, even without pSM [10], 18 of 22 patients with pT3a that had been diagnosed as cT1c or cT2 preoperatively deserved the advantage of RRP-WR because prostate and prostate cancer removal was achieved without pSM.

## Conclusions

To improve prostate cancer control, we performed RRP-WR for 90 cases of preoperative locally confined prostate cancer, and we evaluated the surgical outcome by morbidity and curability with surgical margin status pathologically. We tried to demonstrate preliminary clinical outcomes by achievement of undetectable PSA and BCR, despite the short follow-up period, without immediate adjuvant therapy after surgery.

RRP-WR was as feasible as the conventional procedure, and that surgical outcome, in terms of pSM, was equal or superior to those cases with pT2 and far superior to those cases with pT3 in previous reports. The preliminary clinical outcomes in terms of postoperative undetectable PSA and BCR-free survival without adjuvant therapy encouraged us to continue this procedure for cases preoperatively diagnosed as cT1-2, especially for the cases with high probability of EPE according to the Japan PC Table and cT3a. It is necessary to confirm the long-term results to demonstrate the advantage of this procedure.

## Abbreviations

PSA: Prostate-specific antigen; RRP-WR: Retropubic radical prostatectomy with intentional wide resection; RRP: Retropubic radical prostatectomy; NCCN: National comprehensive cancer network; pSM: Positive surgical margin; EPE: Extraprostatic extension; BCR: Biochemical recurrence.

## Competing interest

The authors declare that they have no competing interest.

## Authors’ contributions

EO designed the study, acquired data, controlled the quality of the procedure as either operator or assistant during surgery for all 90 cases, carried out analysis and interpretation of data, and drafted the manuscript. MY, YM, AI and KaS contributed to the acquisition of data and participated in the surgery as either operator or assistant during some surgeries. HK contributed to the acquisition of data. NT and AH participated in the study design, contributed to the acquisition of data, participated in the surgery as either operator or assistant during some surgeries, and helped to draft the manuscript. KeS made the pathological diagnoses. KF and YH participated in the study design and coordination and helped to draft the manuscript. All authors read and approved the final manuscript.
